# Hepatoprotective Effects of *Silybum marianum* (Silymarin) and *Glycyrrhiza glabra* (Glycyrrhizin) in Combination: A Possible Synergy

**DOI:** 10.1155/2014/641597

**Published:** 2014-03-25

**Authors:** Mahmood Rasool, Javed Iqbal, Arif Malik, Hafiza Sobia Ramzan, Muhammad Saeed Qureshi, Muhammad Asif, Mahmood Husain Qazi, Mohammad Amjad Kamal, Adeel Gulzar Ahmed Chaudhary, Mohammed Hussain Al-Qahtani, Siew Hua Gan, Sajjad Karim

**Affiliations:** ^1^Center of Excellence in Genomic Medicine Research, King Abdulaziz University, Post Box No. 80216, Jeddah 21589, Saudi Arabia; ^2^Department of Pharmacy, University of Lahore, Pakistan; ^3^The Institute of Molecular Biology and Biotechnology, University of Lahore, Pakistan; ^4^Department of Biotechnology and Informatics, BUITEMS, Quetta, Pakistan; ^5^Center for Research in Molecular Medicine, University of Lahore, Pakistan; ^6^King Fahd Medical Research Center, King Abdulaziz University, Jeddah, Saudi Arabia; ^7^Human Genome Centre, School of Medical Sciences, Universiti Sains Malaysia, 16150 Kubang Kerian, Kelantan, Malaysia

## Abstract

Oxidative stress, lipid peroxidation, and transaminase reactions are some of the mechanisms that can lead to liver dysfunction. A time-dependent study was designed to evaluate the ability of silymarin (SLN) and glycyrrhizin (GLN) in different dosage regimens to lessen oxidative stress in the rats with hepatic injury caused by the hepatotoxin carbon tetrachloride. Wistar male albino rats (*n* = 60) were randomly assigned to six groups. Group A served as a positive control while groups B, C, D, E, and F received a dose of CCl_4_ (50% solution of CCl_4_ in liquid paraffin, 2 mL/kg, intraperitoneally) twice a week to induce hepatic injury. Additionally, the animals received SLN and GLN in different doses for a period of six weeks. CCl_4_ was found to induce hepatic injury by significantly increasing serum alanine aminotransferase, aspartate aminotransferase, alkaline phosphatase, and thiobarbituric acid reactive substances while decreasing total protein and the activities of reduced glutathione, superoxide dismutase, and catalase. Treatment with various doses of SLN and GLN significantly reduced ALT, AST, ALP, and TBARS levels and increased GSH, SOD, and CAT levels. Our findings indicated that SLN and GLN have hepatoprotective effects against oxidative stress of the liver.

## 1. Introduction

The liver plays a part in many important functions in the body including metabolism, detoxification, and bile secretion. Additionally, it provides protection from exposure to foreign substances by detoxifying and eliminating them. A healthy liver is very important to overall health because it also handles the metabolism and excretion of drugs from the body [1]. Excessive exposure of the liver to environmental toxins, alcohol, drug overdose, and chemotherapeutic agents such as carbon tetrachloride (CCl_4_) and thioacetamide can damage the liver and cause alcoholic liver disease, followed by hepatitis and cirrhosis.

For centuries, plants and their extracts have been used in the treatment of various human ailments. The secondary metabolites of some plants have antiviral, immunomodulatory, and anti-inflammatory effects on hepatocytes and have proven to be useful in chronic hepatitis. In the last couple of decades, a strong awareness of the safety, efficacy, and cost effectiveness of drug has been developed among the general public, thus increasing the importance and popularity of herbal medicines deemed to be “natural,” as opposed to synthetic drugs [2].

The traditional herbal treatments for liver diseases have reached new heights with the support of modern evidence-based medicines with promising clinical trial results [3].* Silybum marianum* or silymarin (SLN), a plant secondary metabolite, is a complex mixture of four flavonolignan isomers, namely, silybin (60–70%), silychristin (20%), silydianin (10%), and isosilybin (5%) [4, 5]. Although SLN does not have antiviral properties, it has been reported to have antioxidative, anti-inflammatory, immunomodulatory, antilipid, and liver-regenerating properties [1]. The elevated levels of liver enzymes such as aspartate aminotransferase (AST) and alanine aminotransferase (ALT) found in liver injuries and chronic diseases are reduced significantly (30–35%) by its use [6]. SLN has been shown to reduce liver fibrosis up to 30–35%, and in few cases it has reversed the liver fibrosis [7, 8].

Glycyrrhizin (GLN), a glycoside of glycyrrhetinic acid and a plant secondary metabolite, is extracted from the roots of* Glycyrrhiza glabra*, a member of the Leguminosae family. It possesses some nutritive value and medicinal properties [9, 10]. In Japan, GLN is commonly used in the treatment of chronic hepatitis C. GLN significantly reduced plasma ALT and improves liver function in hepatitis C virus infected chronic hepatitis patients [11]. GLN root extract contains saponins, triterpenes, and flavonoids as well as other important constituents, such as phytosterols, choline, and tannins [12–15].

In the laboratory, CCl_4_ is frequently used to induce liver injuries in animals, mimicking the liver damage caused by various hepatotoxins in humans. CCl_4_ generates a highly reactive carbon trichloromethyl radical (CCl_3_) causing hepatocellular necrosis, which also contributes to oxidative stress and lipid peroxidation [16]. Lipid peroxidation, including chloromethylation and saturation, leads to a functional and structural disruption of the unsaturated fatty acids of the membrane phospholipids [17]. Microarray-based whole transcriptome expression studies of CCl_4_-induced rats have found significant changes in the genes involved in stress, DNA damage, cell proliferation, and metabolic enzymes [8, 18, 19]. These profiling studies have established the genetic basis of hepatic toxicity by identifying the molecular responses to acute CCl_4_ toxicity.

The individual hepatoprotective effects of herbs such as SLN and GLN have been investigatedin hepatotoxic damage. However, there are no studies on the hepatoprotective role of these herbs when administered in combination, which may have a synergistic effect against liver damage, particularly against chronic liver hepatitis. In this study, we investigated the hepatoprotective and antioxidative roles of SLN and GLN on CCl_4_-induced liver injury when administered at several different doses, singly or in combination.

## 2. Materials and Methods

### 2.1. Laboratory Animals

Adult Wistar male albino rats ranging from 200 to 250 g were obtained from the National Institute of Health, Islamabad, Pakistan. The animals were fed regular diets and were kept at 25°C with controlled humidity (60%) and lighting (12 h light-dark periods). Water was allowed ad libitum. The study was approved by the Ethics Committee for Scientific Research at the University of Lahore.

### 2.2. Plant Extracts and Chemicals

The standardized extracts of SLN and GLN were purchased from the Sigma-Aldrich Corporation (St. Louis, MO, USA). Ethanol was used as the solvent for SLN and dimethyl sulfoxide was used for GLN. All other chemical reagents were of analytical grades and were also purchased from Sigma.

### 2.3. Induction of Hepatic Damage

A modified model of Yadav et al. (2008) was used. Briefly, a dose of 2 mL/kg (50% solution of CCl_4_ in liquid paraffin) was intraperitoneally administered twice a week to induce hepatic injury in rats [20].

### 2.4. Experimental Design

The rats (*n* = 60) were randomly allocated into six groups. Group A was untreated healthy rats and used as positive control. Groups B, C, D, E, and F received a dose of CCl_4_ twice a week to induce hepatic injury. Group B was injected with CCl_4_ alone without SLN or GLN and was used as negative control. The remaining groups (C, D, E, and F) were treated with different concentrations and combinations of the SLN and GLN extracts and were used as test cohorts:

 Group A = positive control (healthy untreated rats),
 Group B = negative control (CCl_4_ alone),
 Group C = CCl_4_ + SLN (200 mg/kg),
 Group D = CCl_4_ + GLN (50 mg/kg),
 Group E = CCl_4_ + SLN (100 mg/kg) + GLN (25 mg/kg),
 Group F = CCl_4_ + SLN (200 mg/kg) + GLN (50 mg/kg).

The biochemical assays on the rat's blood and liver tissues were conducted at 2, 4, and 6 weeks to confirm the hepatoprotective effects of SLN and GLN.

### 2.5. Blood and Serum Separation

Blood (5 mL) was withdrawn from the rats' tails and the serum was separated by centrifugation for 10 min at 1500 g. The serum was stored at −60°C until further biochemical analysis.

### 2.6. Tissue Homogenate

Liver tissues were homogenized in sodium-phosphate buffer saline (10 mM stock) to yield a 25% homogenate. The homogenate was centrifuged for 15 min at 1500 g and the supernatant was stored at −60°C until the biochemical analysis was performed.

### 2.7. Biochemical Assays

#### 2.7.1. Determination of Liver Enzymes and Total Protein

The ALT, AST, and total protein (TP) levels were determined using commercial kits (Biomerieux, USA) based on the established method [21].

#### 2.7.2. Determination of Alkaline Phosphatase (ALP)

ALP was estimated according to the standard method described by Ochoa (1968) using Randox Kits (Randox Laboratories Ltd., Crumlin, UK) [22].

#### 2.7.3. Estimation of the GSH Content

The content of GSH in the liver was estimated according to the Ellman's method (1959) [23]. Briefly, Ellman's reagent (5,5-dithiobisnitrobenzoic acid) reacts with GSH to produce a chromophore (5-thionitrobenzoic acid acid) and oxidized GSH. First, the sum of the reduced and oxidized GSH based on the chemical formula (GSH) *t* = (GSH) + 2 × (GSSG) was used to measure the amount of GSH in the known samples, where (GSH) *t* = total GSH, (GSH) = reduced GSH, and (GSSG) = glutathione disulfide or oxidized glutathione. Then, a linear equation was generated from several standards of GSH (dynamic range is 0–8 *μ*M GSSG or 0–16 *μ*M GSH) to determine the concentration of an unknown sample [24]. Under the assay conditions, GSSG produced 2 mol equivalents of GSH.

#### 2.7.4. Estimation of the Catalase (CAT) Content

Aebi's method (1984) was utilized for the CAT assay [25]. A neutral phosphate buffer (0.01 M) and hydrogen peroxide (H_2_O_2_) (2 mM) solution was used to homogenize the liver tissue at <4°C. This was followed by centrifugation at 2000 g. The enzyme activity was estimated spectrophotometrically by measuring the decrease in absorbance at 230 nm and was expressed as units/g of liver tissue. The absorbance values (OD) of the reaction mixture containing phosphate buffer, H_2_O_2_, and an unknown quantity of the enzyme extract were then compared with a known standard curve of the CAT. Different volumes (10–150 *μ*L) of the catalase formaldehyde standard (4.25 mM stock solution) were diluted in the buffer to a final volume of 1000 *μ*L to yield a final standard concentration ranging from 5 to 75 *μ*M.

#### 2.7.5. Estimation of the Thiobarbituric Acid Reactive Substances (TBARS) Content

Estimation of lipid peroxidation in liver tissues was colorimetrically determined by measuring the TBARS [26] based on the method established by Ohkawa et al. (1979). Briefly, 0.2 mL of 8.1% sodium dodecyl sulphate, 1.5 mL of 20% acetic acid, and 1.5 mL of 0.8% thiobarbituric acid were added to 0.2 mL of the homogenized sample. It was vortexed for 2 min and then centrifuged at 1500 g for 10 min. The OD of the upper organic layer was then measured at 532 nm. The level of lipid peroxides was expressed as mM of TBARS/100 g of liver tissue.

#### 2.7.6. Estimation of the Super Oxide Dismutase (SOD) Content

The method of Nishikimi et al. (1972), which was later modified and improved by Kakkar et al. (1984), was adopted to measure the SOD activity [27, 28]. Briefly, 1.2 mL of sodium pyrophosphate buffer (pH 8.3, 0.052 M), 0.1 mL of phenazine methosulphate (186 *μ*M), 0.3 mL of nitro blue tetrazolium (300 *μ*M), and 0.2 mL of dihydronicotinamide adenine dinucleotide (NADH) (750 *μ*M) were added to 0.1 mL of the sample. The reaction was initiated by adding NADH followed by incubation at 30°C for 90 sec. The reaction was later terminated by the addition of 0.1 mL glacial acetic acid. Then, 4.0 mL n-butanol was added to the reaction mixture followed by a thorough mixing. The reaction mixture was allowed to stand for 10 min, followed by centrifugation at 1500 g to separate the upper butanol layer from the sample reaction mixture. The presence of chromogen was measured at 560 nm against n-butanol as the control. A standard curve of known SOD concentrations was then used to estimate the unknown SOD concentrations. The standard stock of SOD ranging from 10 to 200 *μ*L was diluted in the buffer to a final volume of 1000 *μ*L to produce a standard with SOD activity ranging from 0.025 to 0.250 U/mL.

### 2.8. Histopathological Examinations

The rats' livers were fixed in 10% formalin for 24 h. This was followed by tap water washing before dehydration using absolute ethyl alcohol. Xylene was used to clean the specimens, which were later embedded in paraffin in a hot air oven at 50°C for 24 h. The processed specimens were fixed in paraffin tissues blocks. A sledge microtome was used to produce tissue sections of 4 *μ*m thickness on glass slides. For histopathological examinations, the slides were deparaffinized and stained with hematoxylin and eosin [29] and examined at various time intervals (2, 4, and 6 weeks).

### 2.9. Statistical Analysis

A CoStat computer package (version 6.4) (CoHort software, Monterey, CA) was used for statistical analysis. The mean ± SEM was used to express the results. A *P* value of <0.05 was considered to be significant.

## 3. Results

### 3.1. The Effects of SLN and GLN on CCl_4_-Induced Changes in Serum ALT, AST, ALP, and TP Levels

A significant increase in the serum levels of the liver enzymes (ALT, AST, and ALP) and a significant decrease in TP levels were observed in all of the animals receiving CCl_4_ alone (Group B) confirming that the dose is adequate and suitable for the induction of hepatic injury (Table 1). Both SLN and GLN, when administered singly (Groups C and D), ameliorated the ALT, AST, ALP, and TP levels indicating that the herbs have hepatoprotective activity even when used alone. When the herbs were used in combination (Group E), the levels were markedly ameliorated indicating that the herbs may have synergistic effects. The highest liver protective effect was observed in Group F, where the animals received the highest dose of the herbs given in combination.

### 3.2. The Effects of SLN and GLN on CCl_4_-Induced Changes in Antioxidant Enzymes (SOD and CAT) Activities in the Liver

CCl_4_ significantly reduced the activity of SOD (from 76.75 to 54.59 *μ*g/mg) and CAT (from 33.16 to 20.25 *μ*g/mg) in the rats' livers. Both SLN and GLN increased the activities of SOD and CAT in all treated groups (C, D, E, and F). Again, the SLN and GLN combination at the higher dose (Group F) yielded the best hepatoprotective effect with an almost 100% recovery (Table 2).

### 3.3. The Effects of SLN and GLN on CCl_4_-Induced Changes in Nonenzymatic Antioxidant (GSH) Activity in the Liver

The activity of GSH significantly decreased (from 7.84 to 2.99 *μ*g/mg or by 61.86%) in the rats' livers following hepatic injury due to CCl_4_ exposure. The highest recovery of GSH (at 7.90 *μ*g/mg protein) was again observed in animals receiving SLN and GLN in combination and the group of animals that received the highest dose (Group F) had the best hepatoprotective effects (Table 2).

### 3.4. The Effects of SLN and GLN on CCl_4_-Induced Changes in the Lipid Peroxidation (TBARS) of the Rats' Liver

An increase of 82.81% in the levels of TBARS (80.51 nmol/g tissue) was recorded in the liver of rats treated with CCl_4_ when compared to that of the normal control animals (44.04 nmol/g tissue) (Table 2). Again, the animals receiving SLN and GLN in combination showed significant recovery of the TBARS levels almost to normal indicating that the herbs may have synergistic effects for liver protection.

### 3.5. Time-Dependent Effects of SLN and GLN

**Figure 1:**
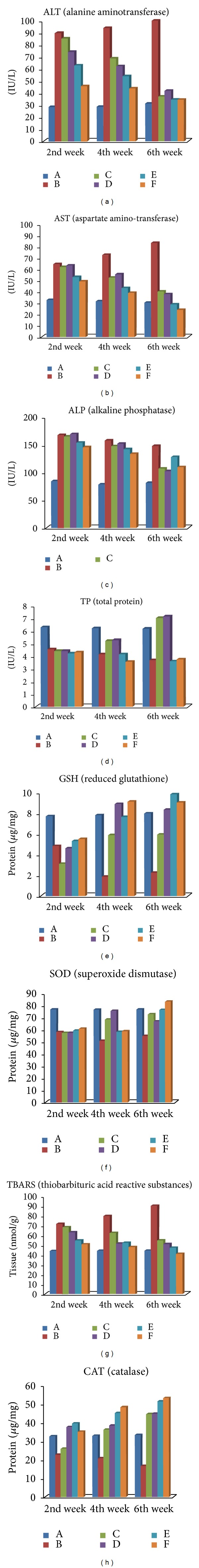
The effects of SLN and GLN in the different groups of rats (ALT, AST, ALP, and TP are expressed as IU/L; GSH, SOD, and CAT are expressed as *μ*g/mg protein; and TBARS is expressed as nmol/g tissue) for the 2nd, 4th, and 6th weeks of treatment.

Overall, SLN and GLN ameliorated the serum levels of the liver enzymes (ALT, AST, and ALP) and TP levels the most when administered for 6 weeks when compared to 4 or 2 weeks (Figure 1). The recovery of ALT (39%, 46%, and 91%), AST (49%, 75%, and 100%), and ALP (27%, 30%, and 65%) continually increased towards normalcy over the time course of the experiment (2nd, 4th, and 6th week). As expected, the highest recovery for the SOD and CAT enzymes (24.16% and 42.12%) was observed in group F at the 6th week following the administration of the herbs. The activity of GSH significantly increased during the 2nd (5.52 *μ*g/mg), 4th (9.15 *μ*g/mg), and 6th (9.05 *μ*g/mg) weeks indicating that SLN and GLN had time-dependent effects in reducing liver damage. Similarly, the TBARS levels were also significantly higher and were almost back to normal by the 6th week of the experiment indicating that by six weeks the maximum hepatoprotective effects were reached by SLN and GLN, especially when administered in combination.

### 3.6. Histopathological Findings

**Figure 2:**
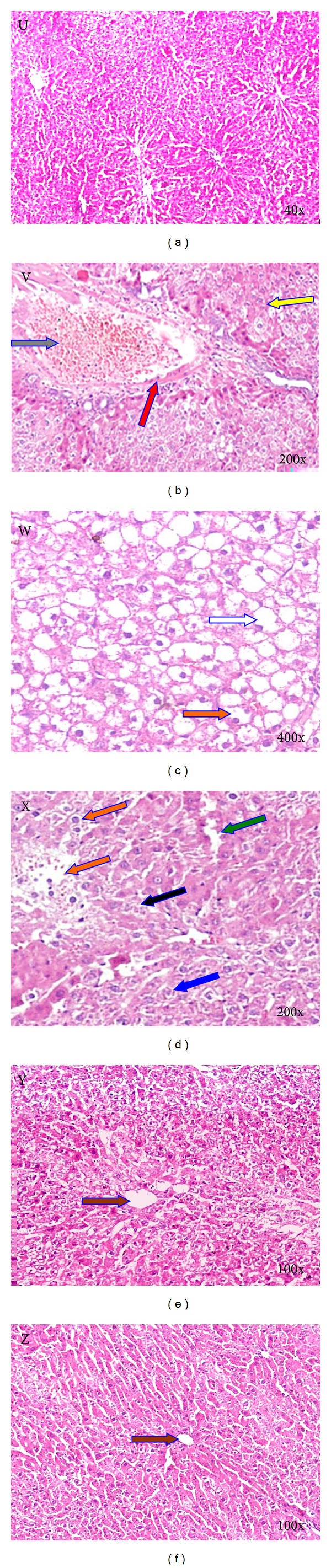
The histological structure of the rat liver. “U” is a normal control; V, W, and X are negative controls representing hepatic injuries at 2, 4, and 6 weeks, respectively; Y and Z are cases representing hepatic injury recovery after 6 weeks of combined SLN and GLN treatment. The arrows represent the status of the cells in different conditions: apoptotic bodies (yellow arrow), degenerated hepatocytes (red arrow), portal area infiltrated by mononuclear inflammatory cellular exudates mainly containing lymphocytes (gray arrow), steatosis (white arrow), binucleated cells (orange arrow), dilated hepatic sinusoids (green arrow), necrosis (black arrow), diffused Kupffer cells (blue arrow), and the central vein (brown arrow).

The histopathological observations (Figure 2) following two weeks of CCl_4_ exposure indicate the presence of liver injury as evidenced by hepatocyte proliferation, necrosis, diffused Kupffer cells, binucleated cells, few mitotic configurations, congestion of central and portal veins ballooning degeneration, and sinusoidal dilation when compared to the animals in the normal control group. These alterations were significantly ameliorated by the combination of SLN and GLN treatments, where only minor hepatocellular necrosis, inflammatory cell infiltration, and mild portal inflammation were observed.

## 4. Discussion

CCl_4_ is widely used to induce liver injury in laboratory rodents [16, 30–32]. Increased levels of serum transaminases reflect hepatic injury as the enzymes are released into circulation following the exposure [33]. CCl_4_ initially causes necrosis and steatosis and may lead to fibrosis, cirrhosis, and hepatocellular carcinoma when administered at higher dosages [34, 35]. Because the changes related to CCl_4_-induced liver injury are in close propinquity to those of viral hepatitis [36], CCl_4_-induced hepatic insult was selected in the current study as the experimental model to investigate the effects of SLN and GLN standardized extracts used singly and in combination at different dosages and over a time course of therapy.

It is widely accepted that, in hepatic parenchyma cells, cytochrome P450-dependant monooxygenases convert the accumulated CCl_4_ into CCl_3_ radicals. In addition to the alkylation of cellular proteins, CCl_3_ attacks the polyunsaturated fatty acids to produce lipid peroxides that are responsible for the hepatotoxicity and alteration of hepatic enzyme levels [37]. The disturbance of hepatocytic transport function during hepatic injury causes an altered permeability of the membrane leading to the leakage of enzymes from the cells [38], thus resulting in the reduction of the ALT, AST, and ALP levels in the hepatic cells and elevation of their levels in the serum [20]. Rajesh and Latha have also shown that cellular leakage and liver cell membrane integrity are linked to increased levels of liver enzymes [39]. ALT and AST are enzymes present in hepatocytes and liver parenchymal cells, respectively. Increased levels of these transaminases are indicators of liver cellular integrity. Large bile duct obstruction and infiltrative diseases of the liver lead to increased ALP levels in plasma because ALP is present in the cells lining the biliary ducts of the liver.

In the present study, CCl_4_ was administered to inflict liver injury and standardized extracts of SLN and GLN were administered to investigate their hepatoprotective effects. CCl_4_ was found to increase the levels of ALT, AST, and ALP while decreasing the TP levels, thus confirming the presence of liver injury, as also reported by Yadav et al. [20]. Interestingly, the animals that received the SLN and/or GLN therapy showed liver recovery that approached normalcy with increasing time courses of administration regardless of whether they were administered singly or in combination.

At the lowest levels of ALT, AST, and ALP for the animals in groups E and F, the combined therapy of SLN and GLN was more effective than when they were used individually and the extract with the higher dose combination was the most effective. The extracts seem to act synergistically to ameliorate the hepatic injuries. In another study, Yadav et al. have also shown that the combination therapy of SLN and* Phyllanthus amarus* extracts has marked hepatoprotective effects as indicated by significant changes in liver enzymes [20]. The drugs in combination produced higher liver protection than when used alone; thus combination therapy can lead to synergistic activity [20, 40]. Our study is the first to show that SLN and GLN have marked hepatoprotective effects when used in combination at 200 mg/kg and 50 mg/kg, respectively.

The overproduction of reactive oxygen species (ROS) in hepatocytes may cause cell death by damaging DNA, proteins, lipids, and carbohydrates [41, 42]. The imbalance between the production of ROS and antioxidant defense causes oxidative stress, leading to significant physiological challenges. The increased levels of TBARS (80.50 nmol/g versus 44.23 nmol/g) and decreased levels of SOD (54.59 *μ*g/mg versus 76.75 *μ*g/mg) and CAT (20.25 *μ*g/mg versus 33.17 *μ*g/mg) in our study suggest that excessive lipid peroxidation results in tissue damage and the failure of antioxidative defenses to mop up the excess production of ROS [43]. The administration of SLN and GLN helped to ameliorate all these cellular changes by increasing the enzymatic antioxidants (SOD, CAT) as well as nonenzymatic antioxidant (GSH) and reducing the TBARS level in the serum. Our findings indicate that SLN and GLN have the ability to scavenge the ROS to overcome the oxidative damage caused by CCl_4_ in artificially induced hepatic injury and that this recovery occurs after six weeks of treatment.

The effects of SLN and GLN were investigated in time-dependent studies at 2, 4, and 6 weeks of treatment to determine when the hepatoprotective effect started to show fully. A continuously decreasing trend in the ALT, AST, and ALP values was recorded after 2nd, 4th, and 6th week in group F rats, which had received a combination of SLN and GLN at the highest dosages. The time-dependent studies revealed that the healing process for serum enzymes induced by SLN and GLN is directly proportional to the time period of treatment. The time-dependent recovery of serum enzymes, SOD, CAT, GSH, and TBARS shows a similar behavior; their maximum recovery was observed after the 6th week of the experiment.

A number of histopathological abnormalities such as cellular necrosis, dilated hepatic sinusoids, degenerated hepatocytes, apoptotic bodies, binucleated cells, focal necrosis, diffuse Kupffer cells, and steatosis are evident in rats receiving CCl_4_ as a hepatotoxin. Portal areas infiltrated by mononuclear inflammatory cellular exudates mainly contain lymphocytes. The results of the current study demonstrate that the combined therapy of SLN and GLN may help in the healing of the necroinflammatory lesions induced by CCl_4_. Previous work by Shalan et al. (2005) and Shaker et al. (2010) clearly demonstrated that SLN has anti-inflammatory potential and can alter histopathological changes induced by CCl_4_, such as ballooning, necrosis, and inflammatory infiltration of lymphocytes [44, 45]; our results confirm these findings.

By definition, synergistic effects can also lead to the enhancement of the bioavailability of one of the extracts if the constituents of one extract affect the others or interact with one another; this can also improve their solubilities [46]. Although it has been hypothesized that the combination therapy of SLN and GLN exhibits synergistic activity and confers higher liver protection, the synergistic effects need to be further confirmed using the Berenbaum method [47]. In follow-up studies, the synergistic effects of SLN and GLN will be further investigated using detailed pharmacological, toxicological, and clinical studies.

## 5. Conclusions

In conclusion, SLN and GLN have hepatoprotective effects against CCl_4_-induced liver injury and are more effective in combination than when used individually. Our findings strongly suggest that the combination of drugs at higher doses, that is, SLN (200 mg/kg) and GLN (50 mg/kg), may have synergistic activity and confer the best hepatoprotective effects. The time-dependent studies revealed that the healing process for serum enzymes induced by SLN and GLN is directly proportional to the time course of treatment and that the herbs achieve an almost complete healing after six weeks of continuous administration.

## Figures and Tables

**Table 1:** The effects of SLN and GLN on liver function.

Group	ALT (IU/L)	AST (IU/L)	ALP (IU/L)	TP (IU/L)
A	29.37 ± 0.77	31.29 ± 0.54	81.54 ± 1.34	6.22 ± 0.03
B	94.83 ± 2.61	73.21 ± 4.44	157.96 ± 4.66	4.12 ± 0.20
C	63.68 ± 11.52	51.49 ± 5.12	139.95 ± 14.11	5.54 ± 0.63
D	59.40 ± 7.72	51.93 ± 6.17	141.41 ± 16.45	5.60 ± 0.66
E	50.42 ± 6.85	41.63 ± 5.73	141.65 ± 6.29	3.98 ± 0.16
F	41.32 ± 2.88	37.19 ± 5.93	129.86 ± 8.76	3.87 ± 0.18

Values are expressed as the means ± SEM; *n* = 10 for each treatment group.

**Table 2:** The effects of SLN and GLN on SOD, CAT, GSH, and TBARS levels.

Group	SOD (*μ*g/mg tissue)	CAT (*μ*g/mg protein)	GSH (*μ*g/mg protein)	TBARS (nmol/g tissue)
A	76.75 ± 0.06	33.17 ± 0.17	7.84 ± 0.06	44.04 ± 0.12
B	54.59 ± 1.71	20.26 ± 1.45	2.99 ± 0.75	80.51 ± 4.35
C	66.24 ± 3.73	35.74 ± 4.37	4.99 ± 0.77	61.83 ± 3.18
D	66.67 ± 4.25	40.41 ± 1.85	7.29 ± 1.09	55.20 ± 3.19
E	64.66 ± 4.80	45.50 ± 2.80	7.60 ± 1.06	51.40 ± 1.78
F	67.68 ± 6.36	45.65 ± 4.39	7.91 ± 0.97	46.56 ± 2.35

Values are expressed as the means ± SEM; *n* = 10 for each treatment group.
